# Identification of *mcr-13* in the Endangered *Lynx pardinus*, and *mcr-1* in *Sus scrofa*, Expands the Diversity of Plasmid-Mediated Colistin Resistance Determinants of *Escherichia coli* from Wildlife

**DOI:** 10.3390/antibiotics15090822

**Published:** 2026-08-24

**Authors:** Pablo Pacheco, Lucía Sánchez, José Luis Píriz, María Gil-Molino, Joaquín Rey, Pedro Fernández-Llario, María Jesús Palacios, Jorge Peña, María Isabel Igeño, Alberto Quesada, Alejandro Gallardo-Soler

**Affiliations:** 1 Departamento de Bioquímica y Biología Molecular y Genética, Facultad de Veterinaria, Universidad de Extremadura, 10003 Caceres, Spain; pablopd@unex.es (P.P.); joseluis.piriz@professor.universidadviu.com (J.L.P.); migeno@unex.es (M.I.I.); alexgsoler@unex.es (A.G.-S.); 2 Innovación en Gestión y Conservación de Ingulados S.L., 10004 Caceres, Spain; luciasg@unex.es (L.S.);; 3 Universidad Internacional de Valencia (VIU), 46002 Valencia, Spain; 4 Unidad de Patología Infecciosa, Departamento de Sanidad Animal, Hospital Clínico Veterinario, Universidad de Extremadura, 10003 Caceres, Spain; mariamilgm@unex.es (M.G.-M.); jmrey@unex.es (J.R.); 5 Instituto de Biotecnología Ganadera y Cinegética (INBIO G+C), Universidad de Extremadura, 10003 Caceres, Spain; 6 Dirección General de Caza, Pesca, Acuicultura, Tauromaquia y Medio Natural Junta de Extremadura, 06800 Mérida, Spain; mariajesus.palacios@juntaex.es (M.J.P.);; 7 Instituto de Productos Cárnicos (IPROCAR), Universidad de Extremadura, 10003 Caceres, Spain

**Keywords:** colistin resistance, *mcr-13*, *Escherichia coli*, wildlife, plasmid-mediated resistance, One Health

## Abstract

**Background**: Plasmid-mediated colistin resistance threatens the effectiveness of one of the last-line antibiotics for treating multidrug-resistant Gram-negative infections. Although wildlife is increasingly recognized as a reservoir of mobile antimicrobial resistance genes, its role in the emergence and dissemination of novel plasmid-mediated colistin resistance (*mcr*) genes remains poorly understood. **Objectives**: This study aimed to characterize two distinct *mcr*-carrying *E. coli* isolates identified in Spanish wildlife. **Methods**: Among 807 faecal samples from healthy wild animals screened, two *mcr*-positive *E. coli* isolates were identified. Genomic characterization included whole-genome sequencing, plasmid analysis, and functional assays, followed by heterologous expression and lipid A characterization by Ultra-High-Performance Liquid Chromatography coupled with High-Resolution Mass Spectrometry (UHPLC-HRMS). **Results**: Analysis of the two *mcr*-positive *E. coli* isolates revealed the first *mcr-1*-positive isolate from a wild boar (*Sus scrofa*) in Extremadura, Spain, carrying *mcr-1* on a 33.3-kb conjugative IncX4 plasmid, while the isolate recovered from an Iberian lynx harbored *mcr-13*, a novel plasmid-borne *mcr*-like gene encoding a putative phosphoethanolamine (pEtN) transferase on a 163-kb non-conjugative plasmid. MCR-13 showed 82% amino acid identity with MCR-10, increased colistin MIC two-fold in a susceptible *E. coli* host, and mediated phosphoethanolamine modification of lipid A, although to a lower extent than MCR-1. **Conclusions**: The co-occurrence of established (*mcr-1*) and a novel *mcr*-like gene *mcr-13*, further supports the role of wildlife as a potential reservoir of emerging resistance genes and highlights the value of including wildlife in One Health surveillance programs.

## 1. Introduction

Colistin (polymyxin E) is a cationic polypeptide antibiotic currently considered a “last-resort” therapeutic option for treating infections caused by multidrug-resistant (MDR) Gram-negative bacteria. Its clinical relevance has re-emerged following the global dissemination of extended-spectrum beta-lactamase (ESBL)-producing and carbapenem-resistant Enterobacteriaceae, particularly *Escherichia coli* and *Klebsiella pneumoniae* [1,2]. Although first isolated from *Paenibacillus polymyxa* subsp. *colistinus* in 1947 [3] and FDA-approved in 1959, its systemic use was historically limited by significant nephrotoxicity and neurotoxicity [4]. However, the scarcity of novel antimicrobial agents against extremely drug-resistant (XDR) pathogens led to its reintroduction into clinical practice in the late 1990s. The widespread, and often unregulated, use of colistin in livestock as a growth promoter has significantly contributed to this crisis, establishing a vast environmental reservoir of resistance genes that link animal health, food safety, and human clinical outcomes [5,6].

The bactericidal mechanism of colistin involves electrostatic interactions with the negatively charged lipopolysaccharide (LPS) of the outer membrane, displacing divalent cations (Ca^2+^ and Mg^2+^) from the phosphate groups of lipid A, thereby disrupting membrane integrity and leading to increased permeability and cell death [7,8]. Resistance typically arises through modifications of lipid A that reduce its net negative charge, decreasing colistin affinity. Historically, this was attributed to chromosomal mutations affecting the PhoPQ and PmrAB two-component systems (TCS) [9,10]. These systems regulate the addition of pEtN and L-4-aminoarabinose (L-Ara4N) to lipid A via the *pmrC* (or *eptA*) gene and *arnBCADTEF* operon, respectively [11,12].

The landscape of colistin resistance changed substantially in 2015 with the discovery of *mcr-1*, the first described plasmid-mediated colistin resistance gene [13]. Unlike chromosomal resistance, *mcr* genes facilitate rapid horizontal gene transfer (HGT) of common plasmid backbones across diverse ecological niches [14]. To date, twelve core *mcr* homologs (*mcr-1* to *mcr-12*) and their variants have been identified globally, predominantly (but not only) in Enterobacteriaceae. Following the discovery of *mcr-1*, its gene family has expanded rapidly. In 2016, *mcr-2* was identified in *E. coli* isolates from diarrheic swine and calves in Belgium [15]. In the following year, the diversity of the *mcr* family increased significantly: *mcr-3* was identified in *E. coli* [16], *mcr-4* in both *E. coli* and *Salmonella* [17], *mcr-5* in *Salmonella* [18], and *mcr-6* within the genus Moraxella [19]. The emergence of novel homologs continued in 2018 with the description of *mcr-7* and *mcr-8* in *Klebsiella* species [20,21]. More recently, these genes further broadened with the detection of *mcr-9* in *Salmonella* in 2019 [22], *mcr-10* in *Enterobacter roggenkampii* in 2020 [23], and *mcr-11* in *Lecrercia* spp. [24] and *mcr-12* in *Pigmentiphaga litoralis* [25] in 2026, underscoring a major public health concern for the management of multidrug-resistant infections.

The epidemiological landscape of *mcr-1* in Spain reveals an early and persistent dissemination across the One Health interface, with retrospective evidence tracing its presence in livestock back to 2004 and in clinical isolates to 2012–2015 [26,27,28]. Despite its established circulation in anthropogenic settings, reports of *mcr-1* in Spanish wildlife remains limited, suggesting an under-monitored reservoir [29,30,31]. Further sporadic detections in wild ungulates (Sus scrofa) and birds of prey—particularly in peri-urban and high-density hunting areas—underscore the spillover risk from livestock and contaminated water sources.

Wildlife species, particularly those inhabiting anthropogenically pressured habitats, act as sentinels for the environmental spread of antimicrobial resistance (AMR), and specifically, the wild boar—owing to its ecological plasticity and frequent presence at the human–livestock–wildlife interface—is a valuable sentinel species for monitoring the spread of these high-priority resistance markers [32]. Moreover, the Iberian lynx, currently the world’s most endangered feline, remains highly vulnerable despite recent population recovery (2663 individuals) [33] due to habitat fragmentation, genetic bottlenecks, and a high dependency on prey populations often subjected to pest control interventions [34]. Monitoring AMR in endangered carnivore species is critical to understanding the spillover of resistance genes into pristine ecosystems.

Here, we report plasmid-mediated colistin resistance determinants in wildlife from Extremadura, southwestern Spain, including the identification of the established *mcr-1* gene and the novel *mcr*-like determinant *mcr-13* in isolates from different wildlife hosts. Together, these findings expand the diversity of plasmid-mediated colistin resistance determinants and support the integration of wildlife into One Health surveillance programs.

## 2. Results

### 2.1. Screening for Colistin-Resistant Strains in Iberian Lynx and Wild Ungulates

Among the 200 bacterial isolates recovered from Iberian lynx faecal samples, a single *E. coli* isolate, designated H1bEc, exhibited the highest colistin resistance found, with a MIC of 1 mg/L. Since this singularity corresponds to the upper limit of the wild-type susceptibility distribution, H1bEc was selected for further analysis, together with Eco134, another *E. coli* strain that was isolated from wild boar and presented true colistin resistance (see below), bacteria underwent phenotypic characterization against a panel of fifteen antimicrobials, remaining susceptible to all tested therapeutic agents (Table 1). To investigate the potential presence of plasmid-mediated colistin resistance determinants, all isolates were initially screened by multiplex PCR using degenerate primers targeting *mcr-1* to *mcr-8,* the only ones known at the time of the study [35]. No amplification products corresponding to known *mcr* genes were detected. Furthermore, Sanger sequencing of the *pmrA* and *pmrB* coding regions revealed no mutations previously associated with colistin resistance [36].

Among the 607 faecal samples collected from wild ungulates across three consecutive hunting seasons, only in one of them from a wild boar was the presence of colistin-resistant *E. coli* detected (prevalence: 0.16%, corresponding to a value in the 0.029–0.927 range of frequencies, with 95% confidence), which was found in the 2021/2022 cohort (*n* = 358). Following the molecular workflow described above, multiplex PCR screening with degenerate primers suggested the presence of a *mcr* homolog [33], which was subsequently confirmed as *mcr-1* through target-specific PCR [13]. This wild boar isolate, designated Eco134, underwent phenotypic characterization against a panel of fifteen antimicrobials. Eco134 displayed a narrow resistance profile, remaining susceptible to all tested therapeutic agents except colistin (Table 1). Broth microdilution assays confirmed colistin resistance, with MICs ranging from 4 to 8 mg/L. Furthermore, Sanger sequencing of the *pmrAB* loci [36] revealed a wild-type sequence lacking resistance-associated mutations, confirming that the colistin-resistant phenotype was exclusively mediated by the acquired *mcr-1* gene. Subsequent active surveillance during the 2022/2023 and 2023/2024 seasons in the same geographical region yielded no further *mcr*-positive isolates.

### 2.2. Identification of a Novel Plasmid-Borne mcr-like (mcr-13) in E. coli

To elucidate the genetic mechanism underlying colistin resistance in the Iberian lynx isolate, the complete genome of *E. coli* H1bEc was sequenced using the Illumina platform. Multilocus sequence typing (MLST) assigned *E. coli* H1bEc to ST394. Screening of the whole-genome sequencing (WGS) data against the ResFinder database (thresholds: 90% identity, 60% minimum length) failed to identify any known acquired resistance determinants, including established mobile colistin resistance genes (*mcr-1* to *mcr-12*). Similarly, re-evaluation of the H1bEc assembly confirmed the absence of resistance-associated polymorphisms within chromosomal genes, including *pmrA*, *pmrB*, *phoP*, *phoQ*, *mgrB*, *pmrD* [10,36,37], the *Kluyvera*-like *arnBCADTEF* operon [38], and the lipid A biosynthesis genes *lpxA*, *lpxB*, *lpxC*, and *lpxD* [39].

**Figure 1:**
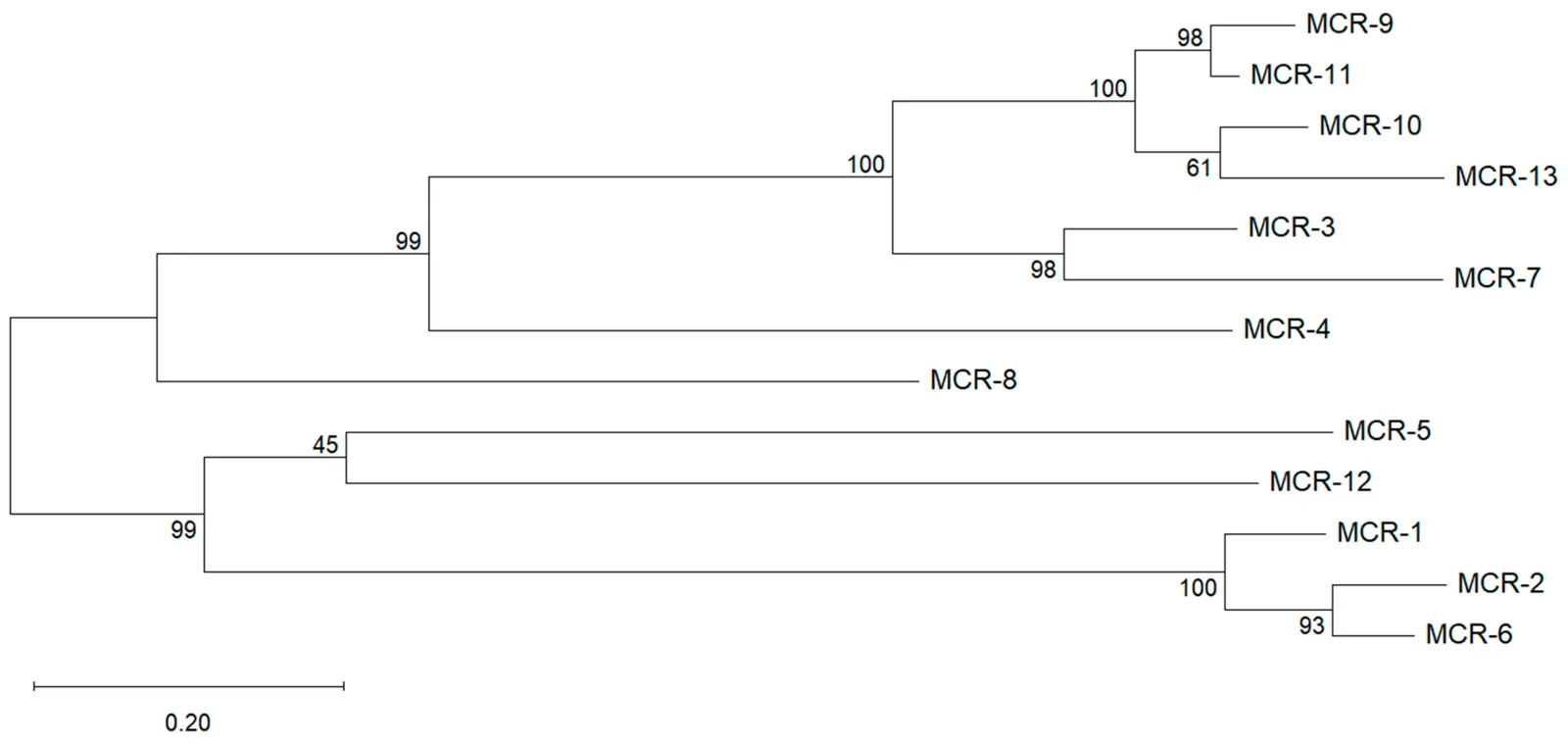
Phylogenetic relationships of plasmid-mediated colistin resistance (MCR) proteins. Protein sequence alignment and phylogenetic tree were generated using MEGA 12.1 [40]. Bootstrap values are indicated and assumed to support the branching of a clade when they are higher than 70% [41], whereas the scale bar represents the genetic distance (0.2 = 20%). Sequences represented are as follows: MCR-1, WP_049589868.1; MCR-2, WP_065419574.1; MCR-3, WP_042649073.1; MCR-4, WP_099156046.1; MCR-5, WP_053821788.1; MCR-6, WP_099982813.1; MCR-7, WP_104009851.1; MCR-8, WP_114699275.1; MCR-9, AYW01299.1; MCR-10, WP_023332837.1; MCR-11, WP_150870284.1; and MCR-12, WP_407792291.1. All sequences shown are from *Escherichia coli* except for MCR-6 (*Moraxella* sp.) and MCR-7, MCR-8, MCR-9 (*Klebsiella pneumoniae*), MCR-11 (*Leclercia* sp.) and MCR-12 (*Pigmentiphaga litoralis*).

Using the PlasmidFinder database, an initial search of the Illumina contigs revealed a single replicon in contig 86 (13,346 bp) sharing 98.66% similarity with an IncX1 plasmid (GenBank accession no. EU370913). By lowering the identity threshold, a second replicon sharing 79% similarity with an IncFIB plasmid (GenBank accession no. AP001918) was identified in contig 69 (25,313 bp). A detailed BLASTn (program version 2.17.0+) analysis of contig 69 revealed a 1626 bp open reading frame (ORF) encoding a putative phosphoethanolamine (pEtN) transferase. This sequence exhibited 99% query coverage and 82% nucleotide identity with *mcr-10.1* (GenBank accession no. NG_066767.1), the closest ortholog among previously characterized *mcr-1 to mcr-12* genes. The encoded 541-amino-acid MCR-13 protein revealed protein identities of 36% (MCR-1), 35% (MCR-2), 67% (MCR-3), 45% (MCR-4), 36% (MCR-5), 33% (MCR-6), 61% (MCR-7), 45% (MCR-8), 77% (MCR-9), 82% (MCR-10), 78% (MCR-11) and 37% (MCR-12). After its phylogenetic clustering, MCR-13 remained positioned within the MCR-9, MCR-10 and MCR-11 clade (Figure 1).

A BLASTn (program version 2.17.0+) search using *mcr-13* as the query identified two almost identical matches with sequences deposited in GenBank but still unpublished at the moment of preparing this manuscript. With due caution regarding sequence files not yet fully consolidated, the first is annotated as a pEtN transferase in the genome of *E. coli* isolated from wild bird faeces, collected in 2018 in New Zealand (accession GCA_017075415.1). The second has already been annotated as MCR-13 and was identified in the genome of *E. coli* from grassland, collected in 2019 in Ireland and deposited in April 2026 (accession JBXAZS010000049.1). Furthermore, in silico screening revealed uncatalogued pEtN transferases (designated as PET-*mcr*) in the genus *Cronobacter* (GenBank accession nos. KAF6590693.1, WP_221413633.1 and WP_283985649.1) and *E. coli* (GenBank accession no. EFF0498833.1) sharing 89% and 84% amino acid identity with MCR-13 respectively. This high degree of sequence conservation points to an uncharacterized reservoir of *mcr*-like determinants within these bacterial groups.

### 2.3. Genomic Architecture and Location of the mcr-13 and mcr-1-Bearing Plasmid

**Figure 2:**
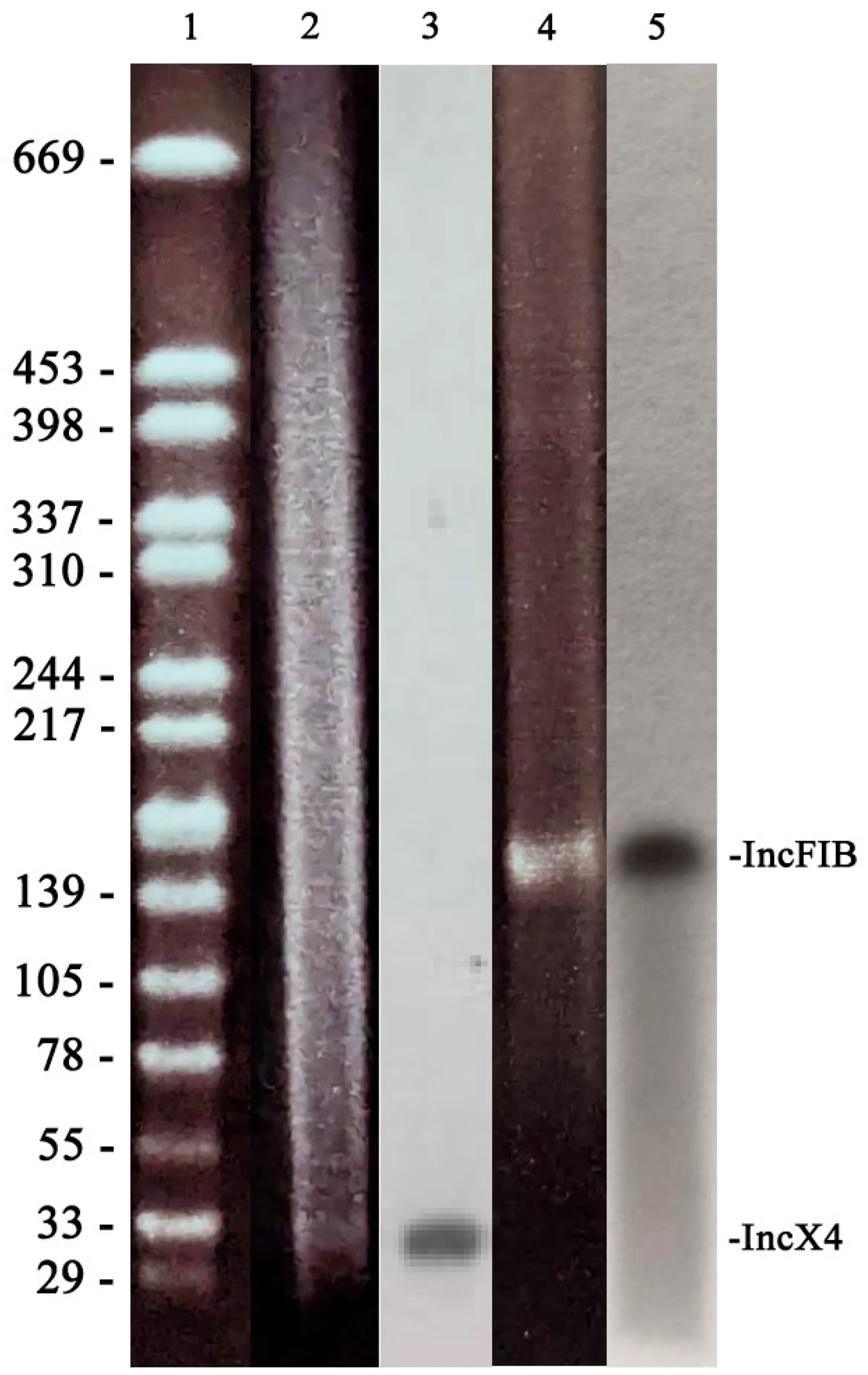
S1-nuclease pulsed-field gel electrophoresis (S1-PFGE) and Southern blot hybridization of *E. coli* Eco134 and H1bEc. Lanes 2 and 4, PFGE digested with S1-nuclease after ethidium bromide staining. Lanes 3 and 5, Southern blot hybridization to DIG-labeled specific probes. Analyzed strains are eco134, lanes 2 and 3 and H1bEc, lanes 4 and 5. *Salmonella enterica* serotype Braenderup digested with XbaI was used as a molecular size marker (lane 1).

Hybrid assembly combining Illumina short reads and MinION long reads revealed that *E. coli* H1bEc possesses a 5,441,270 bp chromosome and harbors two plasmids. The larger plasmid, designated pH1bEc-*mcr-13*, is 163,405 bp in size with a GC content of 49.1% and carries 250 predicted ORFs, including the novel *mcr*-like gene and the IncFIB-type replication origin. The smaller plasmid is 17,157 bp in size with a GC content of 40.97% and contains an IncX1 replication origin (p17linxEc). Both plasmids deviated from the chromosomal GC content (50.5%). The circular topology of both plasmids was verified via PCR-based closure with primers P163kb (CCCGCTGCATTTATATTCCG and GGCCTCTCTTACAGTTTCGT, 554 bp amplification product and 56.8 °C annealing temperature) and P17Kb (CCGTGCTGTATTCATGCATC and GCTATTATTCCGGCAATCCA, 635 bp amplification product and 55.5 °C annealing temperature) and confirmed by Sanger sequencing (Stabvida, Caparica, Portugal). To confirm the plasmid-borne location of *mcr-13*, S1 nuclease digestion followed by pulsed-field gel electrophoresis (S1-PFGE) and Southern blot hybridization was performed. A specific hybridization signal was detected at approximately 160–180 kb (Figure 2), confirming that *mcr-13* is located on the 163,405 bp plasmid. No signal was observed for the p17linxEc plasmid or the chromosome. Despite being located on a plasmid, repeated (twice) conjugation assays failed to transfer pH1bEc-*mcr-13* to the recipient strain *E. coli* J53 using selective media supplemented with sodium azide (100 mg/L) and colistin (2 mg/L), suggesting that this plasmid is non-conjugative under the tested experimental conditions, or that MCR-13 does not promote growth of J53 strain at the threshold for the polymyxin resistance, like in the H1b strain. For the wild boar isolate, S1-PFGE and Southern blot hybridization confirmed that the *mcr-1* gene in Eco134 was localized on a ~30 kb plasmid (Figure 2), and liquid mating assays demonstrated that the *mcr-1*-bearing plasmid, pEco134-*mcr1*, was efficiently transferred to the recipient *E. coli* J53 strain at a high conjugation frequency of 1.3 × 10^−2^ transconjugants per recipient.

### 2.4. Functional Characterization and Lipid A Modification by MCR-13 and MCR-1

To determine whether the novel determinant contributes to colistin resistance, full-length coding sequence of *mcr-13* was cloned into the L-arabinose-inducible expression vector pBAD24 (PCR with primers TGGAGGAATTCATGCCCGTACTATTAAGGA and CAGGTCGACAATCTCTTCAGATGCAGTAAG, 58 °C annealing temperature and 1626 bp amplification product, double digested by EcoRI and SalI) and expressed in *E. coli* J53. Upon induction with 0.2% L-arabinose, *E. coli* J53 harboring pBAD24:*mcr-13* exhibited a colistin MIC of 1 mg/L, representing a 2-fold increase compared to control cells carrying empty pBAD24 vector (MIC = 0.5 mg/L). In comparison, *E. coli* J53 carrying pBAD24:*mcr-1* displayed a colistin MIC of 4–8 mg/L, indicating that MCR-13 confers a lower level of reduced colistin susceptibility than MCR-1. This experiment was performed in triplicate, yielding consistent results.

**Figure 3:**
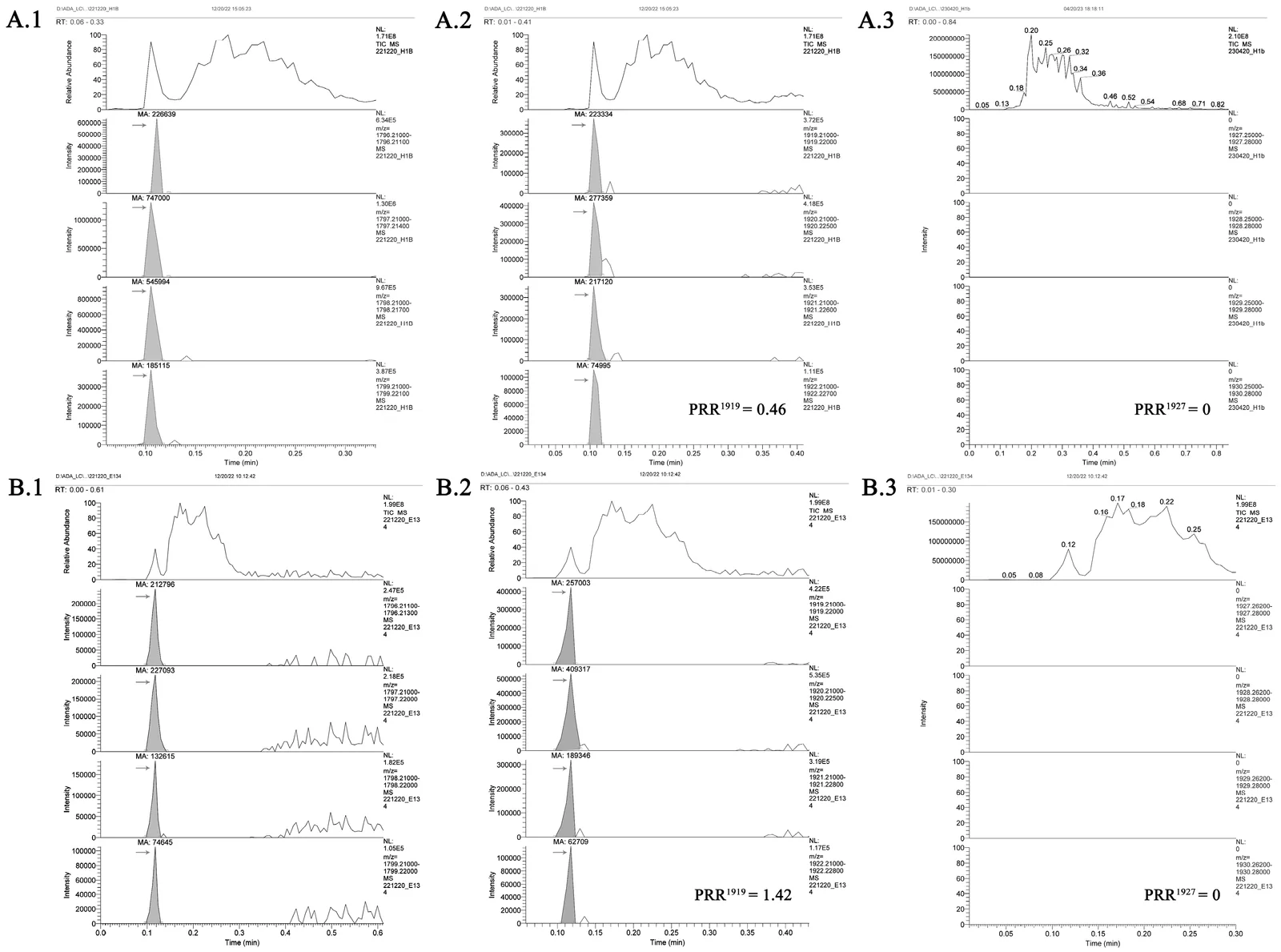
UHPLC-HRMS spectra of Lipid A preparations from *E. coli* strains H1bEc and Eco134. (**A.1**–**A.3**), strain *E. coli* H1bEc; (**B.1**–**B.3**), strain *E. coli* Eco134. Lipid extracts were obtained using the “Blight & Dyer” method (Section 4). The spectra display a signature peak at *m*/*z* ≈ 1919 ((**A.2**) and (**B.2**) for *E. coli* H1bEc and *E. coli* Eco134, respectively), which corresponds to the addition of a PEtN moiety (+123 Da) at the C-1 position of the native lipid, and at *m*/*z* ≈ 1927 ((**A.3**) and (**B.3**) for *E. coli* H1bEc and *E. coli* Eco134, respectively), which corresponds to the addition of an L-Ara4N group (+131 Da) to the 4′-phosphate group. Peak areas (arrows) were integrated to calculate the corresponding PRR (PRR^1919^ = 1919/1796; PRR^1927^ = 1927/1796), considering a maximum of 4 isotopologues (+1 Da relative to the minimum expected value) and only expected *m*/*z* values with an accuracy of ±0.01 Da. PRR^1919^ H1bEc = (223,334 + 277,359 + 217,120 + 74,995)/(226,639 + 747,000 + 545,994 + 185,115) = 0.46; PRR^1927^H1bEc = 0/(226,639 + 747,000 + 545,994 + 185,115) = 0; PRR^1919^Eco134 = (257,003 + 409,317 + 189,346 + 62,709)/(212,796 + 227,093 + 132,615 + 74,645) = 1.42; PRR^1927^Eco134 = 0/(212,796 + 227,093 + 132,615 + 74,645) = 0.

The architecture of lipid A and its link to colistin resistance were characterized via Ultra-High-Performance Liquid Chromatography coupled with High-Resolution Mass Spectrometry (UHPLC-HRMS) [42]. Analysis of the lipid A fraction from *E. coli* H1bEc cultured in medium supplemented with 1 mg/L colistin revealed a distinct mass peak at *m*/*z* 1919.21–1922.21, corresponding to the addition of a pEtN group to the native lipid A backbone. This modification yielded a Polymyxin Resistance Ratio (PRR^1919^) of 0.46 (Figure 3). In comparison, UHPLC-HRMS analysis of Eco134 detected four prominent isotopologues around *m*/*z* 1919.21, resulting in a substantially higher PRR^1919^ value of 1.42 (Figure 3). No L-Ara4N-associated modification to the lipid A structure was detected in either *E. coli* strain H1bEc or Eco134. Consistent with this, a PRR^1927^ of 0 was recorded for both strains. This marked difference in lipid A modification efficiency correlates with the higher colistin resistance level observed in Eco134 (MIC = 4–8 mg/L) relative to H1bEc (MIC = 1 mg/L).

### 2.5. Genetic Context of mcr-13 and mcr-1-Bearing Plasmid

**Figure 4:**
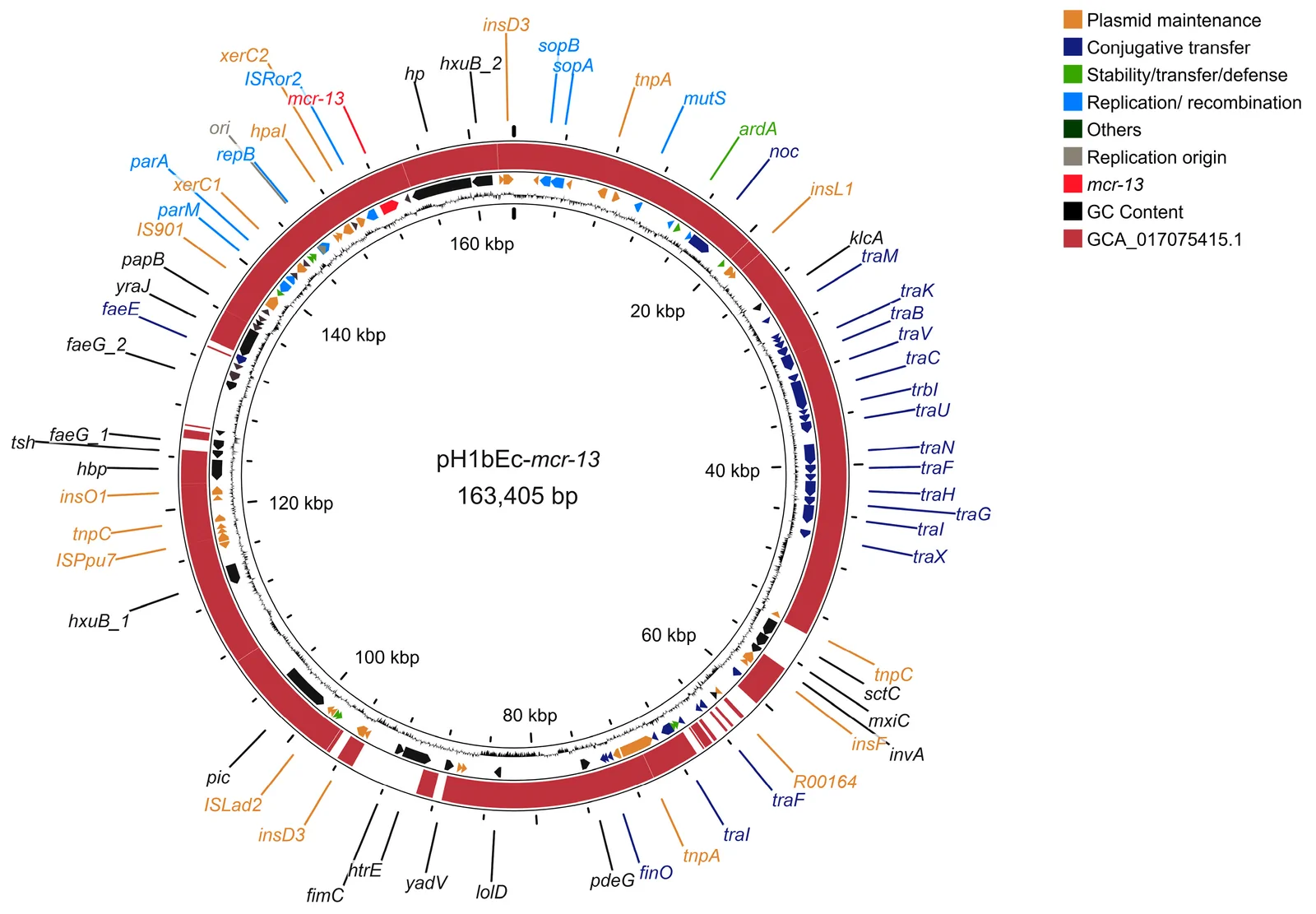
Circular genetic map of pH1bEc-*mcr-13* plasmid predicted with the PROKSEE viewer. Plasmid modules are color-coded as indicated in the top right corner. Hypothetical genes of plasmid pH1bEc-*mcr-13* are omitted for clarity. The blood red ring illustrates the alignment of pH1bEc-*mcr-13* against an isolate retrieved from a New Zealand avian rehabilitation center (GenBank accession GCA_017075415.1).

Comparative genomic analysis of the genetic environment of *mcr-13* within pH1bEc-*mcr-13* plasmid revealed a *xerC* gene positioned immediately adjacent to the resistance determinant. This gene encodes a XerC-type tyrosine recombinase previously associated with recombination and mobilization processes of core resistance fragments, mirroring mechanisms hypothesized for *mcr-10* [22,43,44]. Adjacent to this recombinase, several insertion sequences (*IS*) and transposases (including *insC*, *insD*, and *insE*) were mapped (Figure 4), indicating a region containing several mobile genetic elements potentially involved in mobilization or excision/integration events within the bacterial genome. Additionally, pH1bEc-*mcr-13* encompasses a well-defined transfer (*tra*) region (Figure 4). It primarily consists of genes involved in conjugative processing, including the relaxase *TaxC*, the auxiliary factor *TaxA*, and the coupling protein *TaxB* [45] alongside a transposase (*tnpA*) and a serine recombinase gene (*bin3*) frequently associated with multidrug resistance plasmids [46]. Notably, pH1bEc-*mcr-13* and p17linxEc share an identical 3.7 kb homologous region, representing a potential hotspot for intracellular homologous recombination.

**Figure 5:**
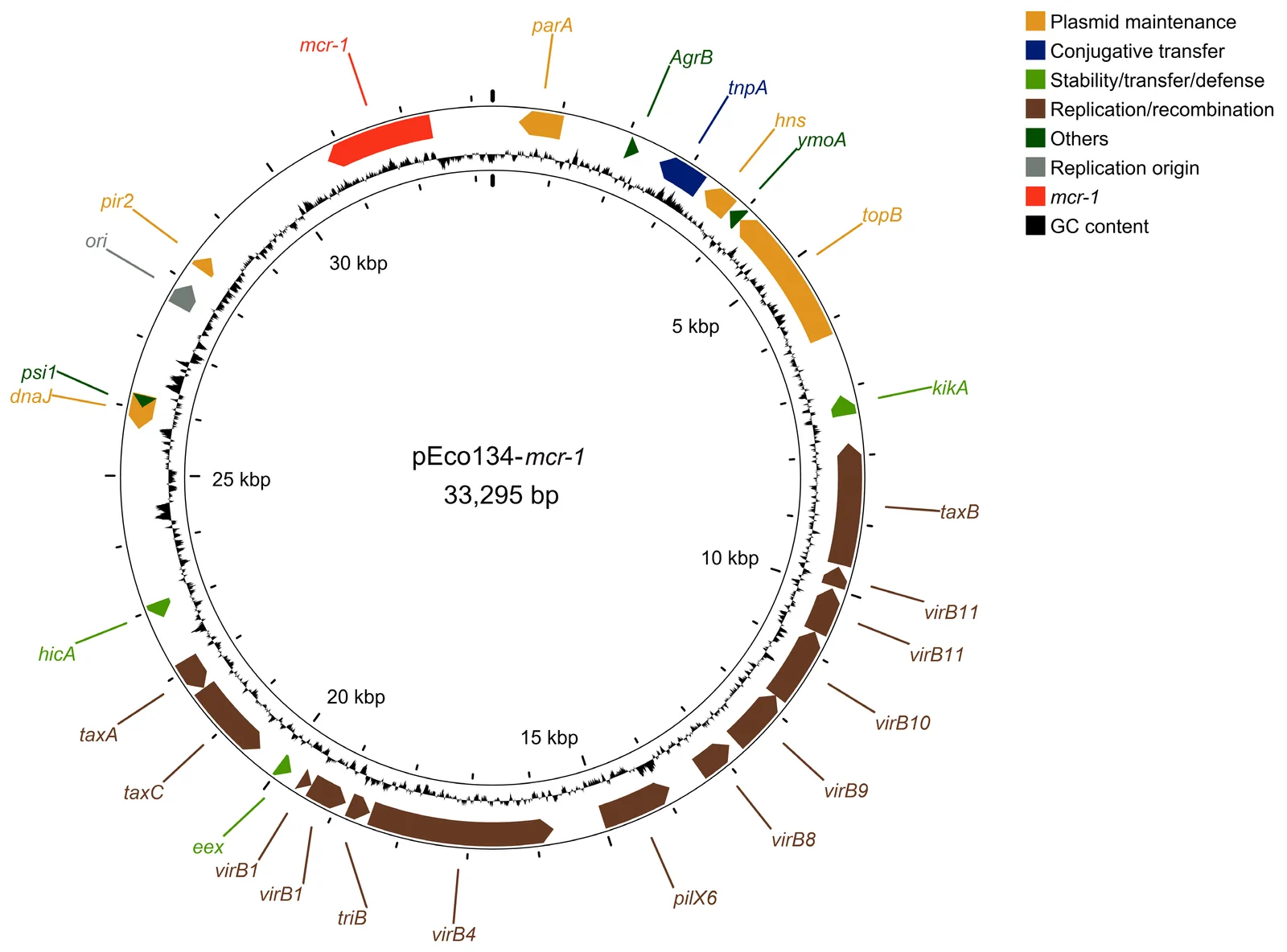
Circular genetic map of pEco134-*mcr-1* plasmid predicted with the PROKSEE viewer. Plasmid modules are color-coded as indicated in the top right corner. Hypothetical genes are omitted for clarity.

For the wild boar isolate, hybrid genomic assembly resolved this element as a 33,295 bp circular plasmid belonging to the IncX4 incompatibility group [47], designated pEco134-*mcr1* (Figure 5). The *mcr-1* gene was flanked by insertion sequence-related features, including a transposase gene (*tnpA*), underscoring its acquisition via horizontal gene transfer rather than as part of the ancestral IncX4 backbone [13]. Notably, no additional antimicrobial resistance genes were detected within the plasmid. Structural analysis revealed a typical IncX4 replication region alongside maintenance and stability determinants, including *pir2*, *parA*, *dnaJ*, and *topB*. The functional partitioning system (*parA*) and toxin–antitoxin modules (*hicA* and *kikA*) likely ensure stable segregation and post-segregational killing, enhancing plasmid persistence without selective pressure. Furthermore, the plasmid harbors a complete type IV secretion system (T4SS)—comprising *taxABC*, the conserved *virB1*-*virB11* operon, and transfer-related elements (*pilX6*, *triB*, *eex*)—confirming its self-transmissibility and potential for horizontal dissemination across bacterial hosts.

## 3. Discussion

The global dissemination of plasmid-mediated colistin resistance (*mcr*) genes represents a critical challenge to the One Health framework, bridging the gap between clinical settings, livestock production, and wildlife reservoirs [48]. While colistin remains a last-resort therapeutic option for treating severe infections caused by multidrug-resistant Gram-negative bacteria [49,50], the emergence of mobile *mcr* homologs threatens to compromise its clinical efficacy. The present study reports the identification of two distinct *mcr* genes in wildlife from southwestern Spain: the first regional identification of *mcr-1* in a wild boar and the discovery of a novel *mcr*-like gene, hereby proposed as *mcr-13*, in an *E. coli* isolate recovered from the Iberian lynx. Both genetic elements have been characterized in parallel by a multiple approach that includes genomics (NGS by short and long reads), biochemistry (Lipid A-directed mass spectrometry) and functional analysis (in vivo plasmid structure and heterologous expression).

The *mcr-1* gene in the wild boar isolate was localized on a 33.3 kb IncX4 plasmid. This specific replicon is globally recognized as a vehicle for the horizontal transfer of *mcr-1* due to its minimized, streamlined genetic architecture and low metabolic burden, which promote stable plasmid maintenance even in the absence of direct antimicrobial selective pressure [51]. High-resolution mass spectrometry of lipid A validated that the phenotypic resistance was mediated exclusively by pEtN modification, correlating with the lack of structural mutations within the chromosomal *phoPQ* or *pmrAB* regulatory systems.

MCR-13 shares between 35% and 82% amino acid identity with previously characterized MCR enzymes, exhibiting the highest degree of protein homology with MCR-10, while remaining sufficiently divergent to constitute a distinct member of the *mcr* family. Phylogenetic reconstruction consistently clustered MCR-13 within the MCR-10 lineage but as a separate branch, supporting its classification as a possible novel homolog. Furthermore, the predicted protein architecture retains the conserved structural features characteristic of pEtN transferases involved in lipid A modification, suggesting conservation of the catalytic mechanism across members of this family. The functional significance of *mcr-13* was further supported by heterologous expression experiments, where its expression in a susceptible *E. coli* host resulted in a reproducible increase in colistin MIC compared to the parental strain. Although the observed increase was modest and did not reach clinical resistance breakpoints, high-resolution mass spectrometry provided additional evidence of lipid A modification at *m*/*z* 1919.22 [42], supporting the hypothesis that MCR-13 functions as a pEtN transferase.

Fitness costs are a known trade-off for plasmid-mediated resistance mechanisms, and reduced growth observed in *mcr-11*-expressing cells may reflect physiological consequences of MCR-mediated outer membrane modifications [51]. In addition, an inducible expression has been described for *mcr-9* [52], which cluster near *mcr-13* in the same *mcr* subfamily (Figure 1). Together, these findings may suggest that *mcr-13* could contribute to low-level colistin resistance and warrant further investigation into its biological significance and regulatory mechanisms.

An additional notable finding was the localization of *mcr-13* on an IncFIB-type plasmid. IncF plasmids are widely distributed among Enterobacterales and play a major role in the dissemination of antimicrobial resistance determinants [53]. The genetic environment surrounding *mcr-13* differed from those commonly described for other *mcr* homologs, suggesting an independent evolutionary trajectory. Although pH1bEc-*mcr-13* failed to conjugate under the experimental conditions tested, its plasmid localization suggests that this determinant may retain the potential for horizontal dissemination under alternative ecological or physiological conditions.

Our findings reinforce the role of wildlife as indicators of environmental antimicrobial resistance circulation [54,55], although the relatively small number of samples examined and the uniqueness of the positive isolates detected limits the scope of the obtained results, the conclusions of which should be considered with appropriate precautions. However, the occurrence of the *mcr-1* gene among ungulates analyzed in this study is extremely low compared to that described in pigs from nearby farms [56], indicating that the transfer of colistin resistance to wild boars is very limited but also suggesting that this animal, which exhibit behavioural adaptations to anthropized environments, is at the potential interface linking livestock (the extensive specially), environmental, and human-associated microbiota, as previously shown with other AMR bacterial populations [32]. In addition, the discovery of the putative new homolog *mcr-13* in the Iberian lynx is noteworthy. Despite its elusive nature and limited direct contact with human activities, this apex predator may become exposed to AMR determinants through the trophic web by preying on wild rodents and lagomorphs that may harbor resistant enterobacteria derived from agricultural runoff or sewage [57]. Furthermore, the presence of uncatalogued pEtN transferases in *Cronobacter* and *E. coli* sharing high amino acid identity with MCR-13 suggests the existence of an under characterized environmental reservoir of *mcr*-like determinants.

## 4. Materials and Methods

### 4.1. Sample Collection and Study Design

A total of 807 faecal samples from wild animals were screened in Spain between 2021 and 2024. The sampling cohort included 607 specimens obtained from wild ungulates (358 in 2021/22, 119 in 2022/23, and 130 in 2023/24), primarily comprising wild boar (*Sus scrofa*, *n* = 568), red deer (*Cervus elaphus*, *n* = 34), and fallow deer (*Dama dama*, *n* = 5). These faecal samples were collected from dead animals during authorized driven hunts across nine Spanish provinces: Badajoz, Cáceres, Cádiz, Ciudad Real, Cuenca, Madrid, Salamanca, Seville, and Toledo. Additionally, a total of 200 faecal samples collected from Iberian lynx subjected to the Strategy for the Conservation of the Iberian Lynx (MITECO, https://www.miteco.gob.es/en/biodiversidad/publicaciones/pbl-fauna-flora-estrategias-lince.html, URL last accessed on 20 August 2026) were processed for bacterial isolation. These faecal samples were isolated between 2019 and 2022 in southern Badajoz, Spain, according to previously published methods [58].

All faecal samples were collected using sterile swabs, placed immediately in Amies transport medium (Becton Dickinson, Franklin Lakes, NJ, USA), and maintained under refrigerated conditions (4 °C) until laboratory processing. In accordance with strict ethical standards, no animals were sacrificed for the purpose of this research, and the authors had no involvement in the death of the animals. All faecal samples were systematically labelled for subsequent culture molecular analyses. For the isolation of colistin-resistant *E. coli*, approximately 100 mg of fecal material was spread into 5 mL of BHI medium (Becton Dickinson) containing a disc with 10 μg of colistin (Becton Dickinson). After overnight incubation at 37 °C, the growth was spread onto McConkey medium (Becton Dickinson) containing 5 μg/mL of colistin. After another overnight incubation at 37 °C, positive colonies were selected for further analysis.

### 4.2. Antimicrobial Susceptibility Testing and Phenotypic Profiling

Bacterial inocula were prepared by overnight growth in Mueller–Hinton II medium (MHII, Becton Dickinson). Antimicrobial susceptibility profiles were determined using the disc diffusion method (Kirby–Bauer) for 15 antimicrobial agents (Table 1), selected according to the European Committee on Antimicrobial Susceptibility Testing [59] guidelines. Inhibition zone diameters were measured and interpreted following the clinical breakpoints established by the Spanish Society of Infectious Diseases and Clinical Microbiology [60]. Isolates were categorized as susceptible (S), intermediate (I), or resistant (R). Multidrug resistance (MDR) was defined as non-susceptibility to at least one agent in three or more antimicrobial classes, as previously described [61]. The reference strain *E. coli* ATCC 25922 was included in each analytical run as a quality control organism to validate disc potency and methodological performance.

For the determination of colistin resistance, the colistin Minimum Inhibitory Concentration (MIC) was determined in milligrams per litre using the broth microdilution method in MH-II (Becton Dickinson) medium according to ISO 20776-1:2019 standards [62]. Colistin resistance was defined based on EUCAST clinical breakpoints. *E. coli* ATCC 25922 was used as quality control.

### 4.3. Screening for Colistin Resistance Determinants: DNA Purification, PCR, and Fragment Sequencing

All isolates potentially compatible with colistin-resistant E. coli were processed for this analysis. Bacterial DNA was extracted using the boiling lysis method from overnight cultures (100 µL) in Luria–Bertani (LB, Becton Dickinson) broth. DNA prepared by same method from *E. coli* ATCC25922 was used for PCR quality control to exclude contamination. Initial screening for mcr homologs (*mcr-1* to *mcr-8*) was performed via multiplex PCR using degenerate primers, as previously described [35], allowing simultaneous detection with a single primer pair. The *mcr-9* and *mcr-10* genes were omitted from this PCR screening because, in contrast to other *mcr* homologs, their contribution to phenotypic colistin resistance is variable and appears to depend on strain-specific regulatory backgrounds. For bacterial isolates yielding an amplicon of the expected size, the specific *mcr* homolog was subsequently identified using homolog-specific primers. Amplifications were conducted using DreamTaq Green PCR Master Mix (2X) (Thermo Fisher Scientific, Madrid, Spain) in an iCycler (Bio-Rad, Hercules, CA, USA), using DNA from verified *mcr-1* to *mcr-5* harboring strains as positive controls. Thermal cycling conditions consisted of an initial denaturation at 94 °C for 5 min, followed by 30 cycles of 95 °C for 30 s, 60 °C for 30 s, and 72 °C for 1 min, with a final extension at 72 °C for 10 min.

Additionally, the complete coding sequences of the *pmrA* and *pmrB* genes were amplified [36] and purified using the MEGAquick-spin Plus Fragment DNA Purification Kit (iNtRON Biotechnology, Seongnam-Si, Republic of Korea) according to the manufacturer’s instructions. Amplicons were resolved by Sanger sequencing (Stabvida, Caparica, Portugal) to identify chromosomal polymorphisms; variants previously reported in colistin-susceptible strains, such as *E. coli* ATCC 25922, were excluded from the analysis [36].

For the *E. coli* isolate harbouring the novel *mcr*-like gene, thermal cycling conditions included an initial denaturation at 94 °C for 5 min, followed by 30 cycles of 95 °C for 30 s, 58 °C for 30 s, and 72 °C for 2 min, with a final extension at 72 °C for 10 min. The primers were designed using Oligo Primer Analysis v.7 software [63] and synthesized by Stabvida (Caparica, Portugal).

### 4.4. Potential for Colistin Resistance Determinant Mobilization (Conjugation Assays)

Conjugation experiments were performed by mating donor cells with the azide-resistant E. coli J53azR recipient strain in LB medium for 20 h at 37 °C, as described previously [38]. Transconjugants were selected on LB agar plates supplemented with 100 mg/L sodium azide and 2 mg/L colistin. Conjugation efficiency was calculated by dividing the number of transconjugants by the total number of donor cells. To confirm successful plasmid transfer, antimicrobial susceptibility testing, specific *mcr*-targeted PCR, and subsequent Sanger sequencing of the amplicons were conducted on the verified transconjugants.

### 4.5. Pulsed-Field Gel Electrophoresis (PFGE), S1 Nuclease-PFGE, and Southern Blotting

Analysis of plasmid profiles was conducted using PFGE (Chef DRII; Bio-Rad, Hercules, CA, USA) following the PulseNet standardized protocol. Total DNA was digested in agarose plugs with S1 nuclease (Thermo Fisher Scientific, Waltham, MA, USA) for 10 min at 37 °C to linearize plasmids. Electrophoresis was performed with pulses ranging from 2.16 to 63.8 s over 21.5 h. *S. enterica* Braenderup XbaI-digested was used as molecular weight standard, and the gels were stained with ethidium bromide. For hybridization, the S1-PFGE gels were transferred to a nylon membrane and hybridized with a digoxigenin-11-dUTP-labeled probe (Roche Applied Science, Mannheim, Germany) generated by PCR using specific primers according to the manufacturer’s instructions (Sigma-Aldrich, St. Louis, MO, USA). PCR conditions included an initial denaturation at 94 °C for 5 min, followed by 30 cycles of 95 °C for 30 s, 58 °C for 30 s, and 72 °C for 1 min, concluding with a final extension at 72 °C for 10 min. The resulting PCR product was purified using the MEGAquick-spin Plus Fragment DNA Purification Kit (iNtRON Biotechnology, Seongnam-Si, Republic of Korea).

### 4.6. Whole-Genome Sequencing (WGS) and Bioinformatic Analysis

Total bacterial DNA was isolated using the Qiagen DNeasy Minikit (Qiagen, Hilden, Germany) according to the manufacturer’s instructions. Short-read whole-genome sequencing was executed using the HiSeq X10 platform (Illumina; San Diego, CA, USA) at Stabvida (Caparica, Portugal). The generated paired-end reads were assembled de novo into contigs using SPAdes v3.13.0. To resolve the complete plasmid architecture, long-read sequencing was additionally performed using a MinION sequencer (Oxford Nanopore Technologies; Oxford, UK) and Flye 2.9.2, respectively (Longseq Applications, Murcia, Spain).

For genomic annotation, Prokka v1.14 [64] and the RAST pipeline [65] tools, combined with BLASTp/BLASTn (program version 2.17.0+) searches in the NCBI database, were employed to annotate plasmid sequences. Circular maps were generated by importing the FASTA sequence into the PROKSEE server [66]. AMR genes were identified from genomic sequences using the ResFinder database (https://genepi.food.dtu.dk/resfinder), while plasmid replicons were typed via PlasmidFinder 2.1 (https://cge.food.dtu.dk/services/PlasmidFinder), and multilocus sequence typing (MLST) was conducted using the MLST server (https://genepi.food.dtu.dk/mlstfinder), which URLs were last accessed on 16 August 2026.

### 4.7. Cloning and Heterologous Expression of mcr-13 in the pBAD24 Vector

To evaluate the functionality of the *mcr-13* gene, a full-length coding sequence was amplified using Phusion Green High-Fidelity DNA Polymerase (Thermo Fisher Scientific, Waltham, MA, USA) from template DNA extracted with the QIAamp DNA Mini Kit (Qiagen, Hilden, Germany). PCR conditions comprised an initial denaturation at 98 °C for 30 s, followed by 30 cycles of 98 °C for 10 s, 58 °C for 30 s, and 72 °C for 2 min, with a final extension at 72 °C for 10 min. The PCR product was purified from a 0.8% low-EEO agarose gel using the MEGAquick-spin Plus Fragment DNA Purification Kit (iNtRON Biotechnology, Seongnam-Si, Republic of Korea), digested with EcoRI and SalI (New England BioLabs, Ipswich, MA, USA), and ligated into the similarly digested pBAD24 (Life Science Market, Nova lifetech Limited, Hongkong) expression vector using T4 DNA Ligase (Thermo Fisher Scientific, Waltham, MA, USA).

The recombinant construct was transformed into chemically competent *E. coli* XL1-Blue MRF′ cells [67], and transformants were selected on LB agar containing 100 mg/L ampicillin. The recombinant plasmid was extracted from three independent clones using the Monarch Plasmid Miniprep Kit (New England BioLabs, Ipswich, MA, USA) and transformed into the *E. coli* J53 recipient strain. The presence of inserts was verified in the transformants via PCR and Sanger sequencing (Stabvida, Caparica, Portugal). Target gene expression was induced in liquid cultures of *E. coli* J53 grown in Mueller–Hinton broth supplemented with 0.2% (*w*/*v*) L-arabinose. The empty vector pBAD24 or carrying *mcr-1* [68], both cloned in the same genetic background, were used as negative and positive controls, respectively. Colistin susceptibility was defined as the highest concentration of antibiotic that yielded visible growth of bacteria, whose values were confirmed using three independent experiments.

### 4.8. Lipopolysaccharide (LPS) Modification Analysis

Lipid A extraction was performed using an overnight culture inoculated from a single colony in LB broth at 37 °C. The optical density (OD_600nm_) was monitored, and 200 mL of fresh broth was inoculated at an initial OD_600nm_ of 0.05 and grown until an OD_600nm_ of 0.8–1.0 was reached. Cells were harvested by centrifugation at 10,000× *g* for 10 min, and lipid A species were isolated according to established protocols [69]. Samples were evaporated under a gentle stream of N2 gas. Characterization of the lipid A species was performed at the Area de Química Sostenible i Energies Renovables, Universitat Rovira i Virgili (Tarragona, Spain) using Ultra-High-Performance Liquid Chromatography coupled with High-Resolution Mass Spectrometry (UHPLC-HRMS) on a Orbitrap ID-X mass spectrometer (Thermo Fisher Scientific, Waltham, MA, USA).

### 4.9. Sequence Data Availability

WGS of strains described in this work corresponds to accessions: *E. coli* H1bEc, JCAIJI000000000 (Nanopore); *E. coli* Eco134, JCAIJJ000000000 (Nanopore); *E. coli* H1bEc, JCAIJK000000000 (Illumina) and *E. coli* Eco134, JCAIJL000000000 (Illumina).

## 5. Conclusions

The co-occurrence of *E. coli* harbouring widely distributed resistance plasmids (*mcr-1*-bearing IncX4) and a novel plasmid-borne *mcr*-like gene (*mcr-13*) underscores the diversity of antimicrobial resistance in wildlife. While *mcr-1* represents the most globally disseminated plasmid-mediated colistin resistance gene, the discovery of *mcr-13* indicates that environmental bacterial populations constitute reservoirs of previously undescribed plasmid-mediated colistin resistance determinants and may represent a previously uncharacterized lineage. Although the current epidemiological distribution and clinical relevance of *mcr-13* remain unknown, its identification expands the known diversity of the *mcr* family and the landscape of plasmid-mediated colistin resistance. Additional investigations are required to fully elucidate its regulatory mechanisms, evolutionary origin, and dissemination potential, underscoring the importance of monitoring wildlife-associated bacterial populations as reservoirs of emerging resistance genes before wider dissemination occurs.

## Figures and Tables

**Table 1:** List of antimicrobials used to test the susceptibility of the *E. coli* H1bEc and Eco134 strains. Antimicrobial susceptibility was interpreted according to the breakpoints established by the Spanish Society of Infectious Diseases and Clinical Microbiology (SEIMC). Inhibition zone diameters are expressed in millimeters (mm).

Antimicrobial Group	Antimicrobial Substance	Quantity (µg)	Inhibition Ø (mm)		Resistant (R)/ Susceptible (S)		MIC Cut-Off (μg/mL)
			H1bEc	Eco134	H1bEc	Eco134	
Aminoglycosides	Gentamicin	10	26	21	S	S	≤4
	Amikacin	30	23	21	S	S	≤16
Cephalosporins	Cefazolin	30	22	25	S	S	≤8
	Cefoxitin	30	20	18	S	S	≤8
	Cefuroxime	30	23	23	S	S	≤4
	Cefotaxime	30	28	33	S	S	≤8
Folate antagonists	Trimethoprim–sulfamethoxazole	1.25/ 23.75	31	28	S	S	≤38/2
Phenicols	Chloramphenicol	30	24	23	S	S	≤8
Penicillins	Amoxicillin–clavulanic acid	20/10	26	19	S	S	≤8/4
	Ampicillin	10	22	19	S	S	≤8
Fluoroquinolones	Ciprofloxacin	5	36	34	S	S	≤1
Tetracyclines	Tetracycline	30	23	20	S	S	≤4
Carbapenems	Imipenem	10	26	29	S	S	≤4

## Data Availability

The data presented in this study are available on request from the corresponding author.
